# Clinical decision support malfunctions related to medication routes: a case series

**DOI:** 10.1093/jamia/ocac150

**Published:** 2022-08-30

**Authors:** Adam Wright, Scott Nelson, David Rubins, Richard Schreiber, Dean F Sittig

**Affiliations:** Department of Biomedical Informatics, Vanderbilt University Medical Center, Nashville, Tennessee, USA; Department of Biomedical Informatics, Vanderbilt University Medical Center, Nashville, Tennessee, USA; Division of General Internal Medicine and Primary Care, Brigham and Women’s Hospital, Boston, Massachusetts, USA; Harvard Medical School, Boston, Massachusetts, USA; Partners eCare, Partners HealthCare, Boston, Massachusetts, USA; Penn State Health Holy Spirit Hospital Medical Center, Camp Hill, Pennsylvania, USA; School of Biomedical Informatics, University of Texas Health Science Center at Houston, Houston, Texas, USA

**Keywords:** clinical decision support, value sets, medication routes, electronic health records, alerts

## Abstract

**Objective:**

To identify common medication route-related causes of clinical decision support (CDS) malfunctions and best practices for avoiding them.

**Materials and Methods:**

Case series of medication route-related CDS malfunctions from diverse healthcare provider organizations.

**Results:**

Nine cases were identified and described, including both false-positive and false-negative alert scenarios. A common cause was the inclusion of nonsystemically available medication routes in value sets (eg, eye drops, ear drops, or topical preparations) when only systemically available routes were appropriate.

**Discussion:**

These value set errors are common, occur across healthcare provider organizations and electronic health record (EHR) systems, affect many different types of medications, and can impact the accuracy of CDS interventions. New knowledge management tools and processes for auditing existing value sets and supporting the creation of new value sets can mitigate many of these issues. Furthermore, value set issues can adversely affect other aspects of the EHR, such as quality reporting and population health management.

**Conclusion:**

Value set issues related to medication routes are widespread and can lead to CDS malfunctions. Organizations should make appropriate investments in knowledge management tools and strategies, such as those outlined in our recommendations.

## BACKGROUND

Clinical decision support (CDS) tools, when implemented well, have been shown to improve the quality and safety of healthcare.^1–3^ In recent years, our team has identified a series of types of CDS malfunctions with a variety of causes, including issues with their conceptualization and build, changes to data nomenclature and codes over time, and electronic health record (EHR) system upgrades.^4–9^

Many types of CDS include medications in their logic,^6^ including CDS tools that suggest prescribing medications, support appropriate dosing, or encourage laboratory monitoring of medications in use. These alerts frequently identify medications using value sets^10^ (the National Library of Medicine [NLM] defines value sets as “lists of codes and corresponding terms, from NLM-hosted standard clinical vocabularies [such as SNOMED CT, RxNorm, LOINC, and others], that define clinical concepts”^11^) or concepts from drug compendia (such as RxNorm or FirstDatabank), including items like generic ingredients and drug classes. For example, an alert that suggests smoking cessation treatment might look for drugs in a “medications used to support smoking cessation” class, or enumerate a list of known smoking cessation aids, such as nicotine lozenges or patches, varenicline, or bupropion. Maintaining value sets such as these is an ongoing knowledge management challenge, as medications are removed from the market or new medications become available over time.

One key descriptor of a medication is its route of administration. Many medications have only a single approved administration route, but others have multiple routes, often with significant differences in clinical indications or monitoring parameters. For example, cyclosporine, when given orally or intravenously, is an immunosuppressive medication used to prevent organ rejection in patients who have had certain solid organ transplants.^12^ When given this way, cyclosporine requires careful monitoring of medication levels. However, cyclosporine is also available in an eye drop formulation which is used to treat dry eyes. When administered ophthalmically, it is minimally bioavailable, and drug-level monitoring is not required. In most drug databases, these different formulations are assigned different codes (eg, eye drops have RxCUI 351291; oral solution is 371667; capsule is 835923), but also have an indicator that they share a common ingredient. When CDS interventions are built that involve medications with multiple routes, CDS builders must determine whether the CDS logic needs to consider the medication route.

Our team has observed and responded to a series of malfunctions in CDS interventions that were built using medication concepts, but not limited by route. These led to false positives (alerts that fired when they should not) as well as false negatives (alerts that did not fire when they should). In this manuscript, we present a case series of CDS malfunctions involving failing to consider medication administration route when it was important to do so. We then make recommendations to reduce these errors.

## METHODS

We reviewed our previously collected database of CDS malfunctions,^6^ as well as more recent malfunctions that have occurred in our organizations, and identified a series of CDS malfunctions related to decision support tools that considered a medication as a data element. Our organizations, together, represent several academic medical centers with both inpatient and outpatient care settings, using several different commercially available EHRs, and several different commercially available drug databases.

## RESULTS

### Malfunction case 1: Alert suggests prescribing a medication to help with smoking cessation fails to fire for patients receiving varenicline nasal spray

Varenicline is an oral medication approved to aid in smoking cessation. It was approved by the FDA for that indication in 2006 under the brand name Chantix.^13^ In 2021, the FDA approved a varenicline nasal spray to treat dry eyes, under the brand name Tyrvaya.^14^ A healthcare organization had a CDS alert which is supposed to fire for patients who are currently smoking and not receiving any smoking cessation medications. The alert logic used a value set of smoking cessation medications which was built using a list of generic drug ingredients, but no route restrictions. The list of generic drugs included varenicline, so when varenicline nasal spray was added to the drug database, it was automatically added to the value set. This caused the alert to be incorrectly suppressed for smokers using varenicline nasal spray for their dry eyes, but who were not receiving other medications to help with smoking cessation. The issue was found when one of the authors happened to see a Facebook ad for Tyrvaya and thought to check his organization’s value sets to see if any of them incorrectly included the nasal medication. The value set was updated, correcting the issue with the alert.

### Malfunction case 2: Alert which suggests ordering a beta-blocker for patients with coronary artery disease or those undergoing coronary artery bypass grafting, fails to fire for patients receiving timolol eye drops

This issue happened at multiple organizations with alerts that suggested giving beta-blockers in certain clinical scenarios (eg, prior to coronary artery bypass grafting, or in patients with coronary artery disease). Most beta-blockers are administered systemically, but timolol is a beta-blocker administered via eye drops and used to treat glaucoma. These organizations had developed value sets of beta-blockers without route restrictions, so they inappropriately included timolol eye drops in addition to the systemic beta-blockers, causing their alerts to not fire in cardiac patients who were taking timolol eye drops but not otherwise taking a systemic beta-blocker. This false negative has the potential to cause underprescription of indicated beta blockers.

### Malfunction case 3: Recruiting tool for study of older adults on multiple antihypertensive drugs, accidentally identifies patients prescribed minoxidil for hair loss

A clinical trial recruitment tool was developed to identify older adults receiving multiple antihypertensive drugs. Minoxidil was originally developed as an antihypertensive drug and is still available in an oral form for this purpose, although the oral form is uncommonly used. However, minoxidil is also available as a shampoo or topical agent for hair regrowth, under the brand name Rogaine. An existing value set of antihypertensive drugs, which included minoxidil, was used for the recruiting tool, and no route restriction was specified, leading to the inclusion of patients using minoxidil for hair loss along with another hypertensive medication. This issue was later discovered and fixed during a subsequent audit of the tool’s logic.

### Malfunction case 4: COVID-19 vaccine scheduling tool erroneously considered patients receiving dexamethasone joint injections to be receiving chronic steroids

Earlier in the COVID-19 pandemic, when vaccine supplies were limited, organizations developed tiered systems for identifying which patients should be offered immunization first. Immunosuppression was a key risk factor, and many organizations prioritized giving COVID-19 vaccinations to immunosuppressed patients. One cause of immunosuppression is ongoing usage of steroids, especially at higher doses. Steroids are available in a variety of routes, including oral steroids, injectable steroids, eye drops, and ear drops. Patients often receive intra-articular joint injections of steroids to treat joint pain. One organization discovered that their CDS tool for COVID-19 vaccination prioritization did not differentiate between intravenous and intramuscular injections of steroids (which are systemically available) and intra-articular injections, which tend to stay local and do not induce much immunosuppression, due to an issue with missing route specification.

### Malfunction case 5: Alert incorrectly suggests drug-level monitoring for patients receiving ophthalmic cyclosporine

As described in the background, cyclosporine, when used for organ transplantation, requires regular monitoring. We previously described a case where an alert recommended monitoring for patients receiving cyclosporine eye drops, where such monitoring is unnecessary.^4^ This malfunction was found through comments from users, who overrode the alert, providing comments like “cyclosporine is eye drops!” and “stupid EPIC reminder-N/A for ophthalmic CyA.” The alert was modified to include only systemic forms of cyclosporine, and the problem was resolved.

### Malfunction case 6: Duplicate alert fires when prescribing a systemic steroid when the patient is receiving a topical steroid

Patients who require systemic steroids may already be prescribed topical or ophthalmic steroid preparations for unrelated reasons. In some EHRs, this may trigger unhelpful duplicate medication alerts. Such false-positive alerts annoy users, contribute to alert fatigue, clinician burden, and ultimately clinician burnout.^15^

### Malfunction case 7: Failure to trigger an alert when prescribing an injectable anticoagulant for a patient already on an oral agent

An organization had duplicate therapy alerts which fired for patients receiving multiple anticoagulants. However, the alert was configured to not alert on duplicates with different routes of administration, so patients receiving both oral and injectable anticoagulants did not get the alert, even though this duplication was likely to be inappropriate. Although it may make sense to suppress duplicate therapy alerts involving different routes of administration in many cases (eg, ketoconazole shampoo and oral ketoconazole can be safely coprescribed), there are cases, such as anticoagulants, where the alert should still fire, so this configuration should be handled at the level of the drug rather than as a single setting for the entire EHR.

### Malfunction case 8: Alert which incorrectly suggests QTc monitoring for patients receiving ophthalmic fluoroquinolones

Patients with baseline prolonged QTc (QT interval corrected for heart rate) intervals on their electrocardiogram are at risk of developing ventricular arrhythmias when given medications that can prolong their QTc further. An alert was developed to warn clinicians when prescribing these medications. This alert inadvertently fired for patients taking ophthalmic fluoroquinolones, which do not have the same risk of QTc-prolongation as systemic fluoroquinolones. This was discovered through comments from users who overrode the alert. The alert was modified to only include systemic forms of these medications.

### Malfunction case 9: Alert which discourages duplicate anesthetic administration inadvertently fires for topical anesthetics

Patients who receive liposomal bupivacaine are at risk of adverse effects when additional anesthetics are given within 96 h of the bupivacaine, and an alert was developed to prevent this subsequent administration. Some medications that should be avoided include lidocaine, which can be given via multiple routes, including topically, which does not have the same risk of adverse events as other routes, but was still included in the value set. This was discovered through comments left by users who disagreed with the alert. The alert was modified to exclude topical administrations of lidocaine.

## DISCUSSION

CDS malfunctions associated with missing logic related to administration routes are common and can affect the accuracy of CDS interventions. Inaccurate alert firings can result in suboptimal care and are also a source of user frustration.^4^

Several strategies can be used to avoid or detect CDS logic involving route of administration errors:

1. When CDS developers are designing and building CDS interventions that use medication concepts, they should consider whether a route restriction is needed for each medication concept. For example, it is frequently appropriate to restrict logic to systemically available routes. This limitation can even be considered for medications that do not currently have any nonsystemically available routes, as new dosage forms may be added in the future (as in the case of varenicline)
2. CDS developers should audit existing CDS intervention logic and value sets to identify those that do not currently evaluate route of administration and consider adding a route limitation where appropriate. We have found that value sets and alerts that contain both a systemically available and nonsystemically available form of the same medication are frequently erroneous, so running a query to identify these potentially erroneous situations is high yield
3. Since new dosage forms and routes of administration may become available for existing drugs, knowledge engineers should consider creating reports to monitor for the availability of new routes for existing drugs, especially those used in value sets and CDS tools. When a new route is first seen, relevant value sets and CDS tools can be assessed to determine if changes to the logic are needed. New medications are often added to EHR drug databases on a daily or weekly basis, so ideally an automated job would notify knowledge engineers whenever a new drug is added to the system, flagging those that represent new routes for existing drugs
4. Developers of medication knowledge bases, such as RxNorm or commercial compendia, should be thoughtful about how to handle routes. For example, in RxNorm, drugs are mapped to classes and indications at the level of the IN (ingredient) term type. IN does not include a route, so it is difficult in RxNorm to capture route-specific indications, and value sets at the IN level will intrinsically contain all routes of administration for the included ingredients
5. Finally, user feedback tools for CDS can also identify administration route issues, and knowledge engineers reviewing feedback should consider whether negative user feedback could be drawing attention to a route issue.

It is worth noting that our analysis focused on CDS malfunctions, but medication value sets are frequently used in other contexts, such as reporting, analytics, and data display. These same value set issues may cause patients to be incorrectly included or excluded from reports, quality measure calculations, or population health management tools. Organizations should monitor and manage all value sets, not just those used in CDS.

We also note that, in some cases, the medication route acts as a proxy for the indication of use for the medication. Structured capture of indications at the time of order entry could also be useful for CDS and quality measurement, though it is challenging.^15^

Although the organizations from which these cases were drawn were diverse, a limitation of our study is that there may be other classes of route-related CDS malfunctions that did not occur in our settings, but could occur elsewhere, so those seeking to improve medication-related CDS should consider route-related issues (and other medication-related challenges) broadly to ensure quality CDS.

## CONCLUSION

Value set issues related to medication routes are widespread and can lead to CDS malfunctions. Organizations should make appropriate investments in knowledge management tools and strategies, such as those outlined in our recommendations.

## FUNDING

Research reported in this publication was supported by the National Library of Medicine of the National Institutes of Health under Award Number R01LM011966 and R01LM013995. The content is solely the responsibility of the authors and does not necessarily represent the official views of the National Institutes of Health.

## AUTHOR CONTRIBUTIONS

Drs AW and DFS made substantial contributions to the conception and design of the work. All authors assisted in the acquisition, analysis, and interpretation of data. Dr AW wrote the initial draft, and all authors revised it critically for important intellectual content. All authors had final approval of the version to be published, and all agree to be accountable for all aspects of the work in ensuring that questions related to the accuracy or integrity of any part of the work are appropriately investigated and resolved.
